# An AI Approach to Identifying Novel Therapeutics for Rheumatoid Arthritis

**DOI:** 10.3390/jpm13121633

**Published:** 2023-11-23

**Authors:** Jency R. Rajan, Stephen McDonald, Anthony J. Bjourson, Shu-Dong Zhang, David S. Gibson

**Affiliations:** 1 Personalised Medicine Centre, School of Medicine, Ulster University, Londonderry BT47 6SB, UK; rajan-j2@ulster.ac.uk (J.R.R.); aj.bjourson@ulster.ac.uk (A.J.B.); sd.zhang@ulster.ac.uk (S.-D.Z.); 2 Rheumatology Department, Altnagelvin Hospital, Western Health and Social Care Trust, Londonderry BT47 6SB, UK; stephen.mcdonald@westerntrust.hscni.net

**Keywords:** rheumatoid arthritis, drug repurposing, connectivity mapping, transcriptomics

## Abstract

Rheumatoid arthritis (RA) is a chronic autoimmune disorder that has a significant impact on quality of life and work capacity. Treatment of RA aims to control inflammation and alleviate pain; however, achieving remission with minimal toxicity is frequently not possible with the current suite of drugs. This review aims to summarise current treatment practices and highlight the urgent need for alternative pharmacogenomic approaches for novel drug discovery. These approaches can elucidate new relationships between drugs, genes, and diseases to identify additional effective and safe therapeutic options. This review discusses how computational approaches such as connectivity mapping offer the ability to repurpose FDA-approved drugs beyond their original treatment indication. This review also explores the concept of drug sensitisation to predict co-prescribed drugs with synergistic effects that produce enhanced anti-disease efficacy by involving multiple disease pathways. Challenges of this computational approach are discussed, including the availability of suitable high-quality datasets for comprehensive analysis and other data curation issues. The potential benefits include accelerated identification of novel drug combinations and the ability to trial and implement established treatments in a new index disease. This review underlines the huge opportunity to incorporate disease-related data and drug-related data to develop methods and algorithms that have strong potential to determine novel and effective treatment regimens.

## 1. Introduction

Rheumatoid arthritis (RA) is a systemic and chronic autoimmune disorder that affects musculoskeletal joints, resulting in persistent synovitis, hyperplasia, autoantibody production, cartilage and joint destruction, erosion, and functional impairment [1]. Extra-articular involvement of other organs in RA frequently results in dermatological, neurological, cardiovascular, pulmonary, renal, and gastrointestinal pathology [2].

RA has a global prevalence of 0.24–1%. It has a prevalence rate of approximately 1% in the UK, with an incidence of 1.5 and 3.6 per 100,000 in men and women, respectively, indicating a predilection towards women [3,4]. It is estimated that over 450,000 adults in the UK have rheumatoid arthritis [5]. The disorder can occur at any age, but the average age of onset is between 30 and 50 years [6].

Multiple genetic factors interacting with the environment have been implicated in the susceptibility to and pathogenesis of RA. Alleles of the highly polymorphic human leukocyte antigen (HLA) gene, particularly the HLA-DRB1 gene, have been associated with an increased risk of developing the disorder [7]. The presence of specific HLA-DRB1 alleles containing the shared epitope is known to contribute to the aberrant immune response and the production of autoantibodies, including rheumatoid factor (RF) and anti-citrullinated protein antibodies (ACPA) [8]. Genome-wide association studies have identified many non-HLA loci associated with RA susceptibility. Several well-established gene associations are PTPN22, CTLA4, STAT4 and PADI4 [7]. Polymorphism in the PTPN22 gene is one of the strongest associations of a non-HLA gene to the development of the disorder thought to influence the immune activation threshold of T cells and B cells [9]. CTLA4 genetic polymorphisms may influence T-cell activation and the balance between regulatory T cells and effector T cells, thus affecting immune tolerance [10]. STAT4 is involved in the regulation and promotion of pro-inflammatory cytokines, such as IL-12, IL-23 and type 1 interferon, which contribute to chronic inflammation in RA [11]. Studies have found that PADI4 gene variants contribute to the production of ACPA, leading to the progression of joint inflammation and destruction [12]. Environmental risk factors influencing the development of RA include smoking, air pollution, obesity, occupational exposures such as silica and textile dust, infections, vitamin D deficiency, immunisations, oral contraceptives, and socioeconomic status [7,13,14,15,16,17]. Epigenetic factors are understood to play a pivotal role in the pathogenesis of RA, contributing to the dysregulation of gene expression observed in this autoimmune disorder. DNA methylation alters gene silencing patterns, impacting immune regulation in RA. Histone modifications, like acetylation, methylation and citrullination, affect chromatin structure and gene transcription, increasing expression of genes linked to inflammation and autoimmunity [18]. The dysregulation of non-coding RNAs, particularly microRNAs (miRNAs), exert post-transcriptional control over gene expression, influencing immune response and contributing to joint damage.

Clinical manifestations of the disorder include symmetrical small joint pain and swelling, predominantly of the hands and feet, with associated early morning stiffness lasting for >30 min and often leading to limited function and mobility [19]. Further features may include but are not limited to rheumatoid nodules, tenosynovitis, rashes, fever, and weight loss [20]. Early diagnosis within an optimal therapeutic window of between 3 and 6 months is identified to be key in achieving the most desirable and cost-effective outcome [21]. Early diagnosis and management are vital for initiating timely intervention to suppress inflammation before joint damage occurs and improve the quality of life for individuals with RA. This poses a challenge as it focuses heavily on clinical evidence obtained from the patient’s medical history and physical assessment accompanied by blood tests and imaging studies. Physicians evaluate a patient’s medical history and conduct a thorough physical examination to assess joints using diagnostic criteria based on the 2010 American College of Rheumatology (ACR)/European League Against Rheumatism (EULAR) classification criteria. Serologic blood tests are used to detect RA-specific biomarkers, such as RF and ACPA [22]. Additionally, the severity of disease and inflammation can be assessed using blood tests for inflammatory markers, such as C-reactive protein (CRP) and erythrocyte sedimentation rate (ESR). Imaging techniques, such as X-rays and ultrasound, are used to evaluate the extent of joint damage and monitor disease progression [23].

Currently, treatment and management of RA are aimed at gaining control of inflammation and alleviating pain while maximising joint function with the long-term goal of achieving remission or low disease activity. Treatment plans usually consist of medications, exercise, physiotherapy and occupational therapy [19]. These treatment plans are tailored to each patient according to their age, current disease state, occupation, compliance, and overall health [19]. Treat-to-target (T2T) is a medical strategy that was established in 2010 to guide clinicians to help patients achieve the goal of clinical remission or low disease activity. It advocates early diagnosis and concomitant therapy implementation, followed by regular monitoring of disease activity and adaptation of therapy accordingly, as well as providing education and support for self-management [21,24].

**Figure 1:**
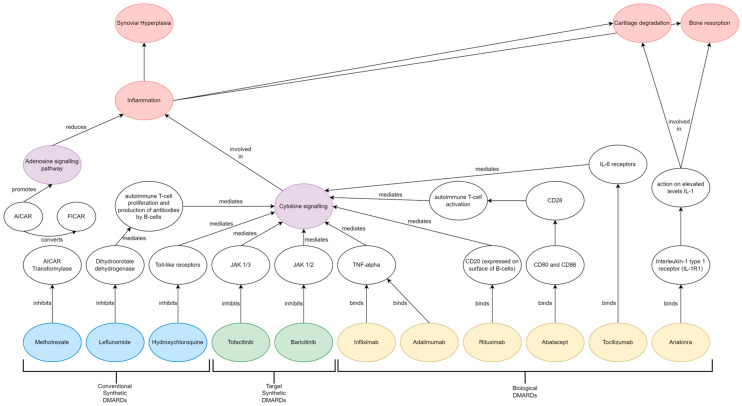
DMARDs used in treatment of rheumatoid arthritis (**bottom** row) with drug class (labelled brackets) and overview of their mechanism of action in terms of known molecular targets (**middle** white ovals) and impact upon cellular pathology (**upper** coloured ovals).

**Figure 2:**
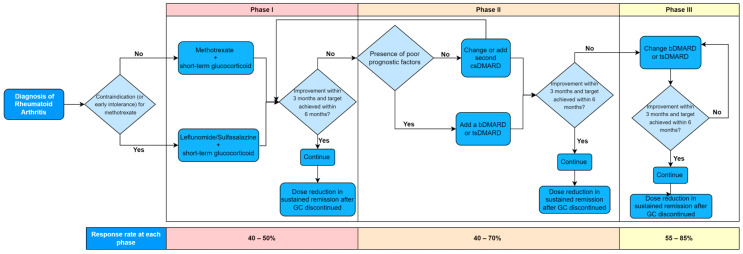
Treatment algorithm adapted from 2022 EULAR rheumatoid arthritis (RA) management recommendations [27]. The average rate of response to administered treatment at each phase is shown below algorithm.

The main drug classes include conventional synthetic disease-modifying anti-rheumatic drugs (csDMARDs), biological DMARDs (bDMARDs) and target synthetic DMARDs (tsDMARDs) (Figure 1). Figure 2 depicts the treatment algorithm based on the 2019 update of the EULAR recommendations on RA management, allowing sequential use of drugs from the main classes [25]. Recommendations are separated into three phases, with regular monitoring of disease activity at 3-month intervals. Treatment is continued if there is improvement at 3 months and the target is achieved at 6 months, followed by potential dose reduction in sustained remission. No improvement results in progression to the next phase of treatment. Phase I treatment recommends methotrexate or leflunomide and short-term glucocorticoids to combat the inflammation. Phase II recommends the change or addition of a second csDMARD or, in the presence of poor prognostic factors, the addition of a bDMARD or JAK inhibitor. Phase III recommends a change in bDMARD or JAK inhibitor. The 2020 NICE guidelines offer a similar trial-and-error treatment algorithm for healthcare providers to follow in a clinical setting [26].

Non-steroidal anti-inflammatory drugs (NSAIDs) and corticosteroids are initially administered to decrease inflammation and alleviate the pain and stiffness in the joints [28]. However, NSAIDs are reported to increase the risk of gastric damage and cardiovascular disease [29]. Although EULAR recommends the use of corticosteroids in combination with DMARDs immediately after diagnosis, and they are frequently continued longer term at a lower dose to retain control of inflammation, there is limited clarity regarding their effectiveness and safety for the treatment of RA to justify extended use [30]. Results of the SEMIRA trial involving patients in remission and treated with conventional synthetic DMARDs in combination with glucocorticoids for more than 6 months showed approximately two-thirds of patients achieved treatment success within 24 weeks once tapered off glucocorticoids. This suggests that discontinuation or tapering of glucocorticoids should be considered for patients in remission and only reintroduced in the case of a flare [30].

Methotrexate, leflunomide and sulfasalazine are the most common csDMARDs used as first-line therapy, administered both as monotherapy and in combination with other DMARDs. Methotrexate is a folate antagonist that functions by inhibiting 5-aminoimidazole-4-carboxamide ribonucleotide (AICAR)-transformylase from converting AICAR to 5-formyl-AICAR (FICAR), which increases adenosine levels and initiates an anti-inflammatory state [30,31]. Leflunomide, on the other hand, functions by inhibiting dihydroorotate dehydrogenase and tyrosine kinase essential for pyrimidine synthesis via *de novo* pathway and therefore inhibiting autoimmune T cell proliferation and B cell autoantibody production [32]. Sulfasalazine is another anti-rheumatic drug where the mechanism of action is not fully understood; however, it is postulated to regulate osteoblasts and osteoclasts by inhibiting NF-kB ligand (RANKL) expression [6]. Common side effects of csDMARDs include gastrointestinal problems, headache, nausea, alopecia, and deranged liver function tests [33]. Folic acid supplementation is shown to reduce the risks of adverse events due to methotrexate therapy [34]. It is estimated only 40–50% of patients achieve a good response to csDMARD treatment [35]. A study conducted in China reported that treatment with methotrexate, leflunomide and sulfasalazine resulted in 39.6%, 33.7% and 48.6% of patients, respectively, experiencing adverse events [33].

bDMARDs are a class of highly effective drugs with a targeted mechanism of action that can rapidly reduce the progression of joint destruction [19]. Infliximab and adalimumab are recombinant monoclonal antibodies that bind to all forms of TNF-α and inhibit inflammatory cytokine production and apoptotic pathway initiation [36]. Rituximab is a monoclonal antibody that binds to presenting CD20 antigens on the B-cell surface and activates the complement system, which eliminates the B-cells from blood circulation. Tocilizumab is a recombinant monoclonal antibody that functions as an IL-6 receptor antagonist, thereby inhibiting IL-6-mediated signalling and expression of pro-inflammatory cytokines [37]. Side effects of bDMARDs are dependent on the mechanism of the drug employed but may include increased infection, mucocutaneous reactions, hypercholesterolaemia, nasopharyngitis and gastrointestinal perforations [38]. It is estimated only 40–50% of patients achieve a good response to bDMARD treatment [35].

tsDMARDs are a relatively novel drug class of orally administered small molecules targeted at intercellular kinase and phosphodiesterase inhibitions. Janus-Kinase (JAK) inhibitors are a class of tsDMARDs that interfere with the ATP-binding sites of JAKs, resulting in the suppression of downstream signalling pathways, which can have immunomodulatory effects in a wide range of pathological processes [39]. Tofacitinib, baricitinib and upadacitinib are approved by the FDA for therapeutic use in RA management [39]. JAK inhibitors are reported to have achieved greater improvements in pain when compared to anti-TNF treatments with a similar mechanism of action [40]. Side effects of JAK inhibitors may include hyperlipidaemia, viral infection, rashes and occasionally an elevated risk of cardiovascular events and venous thromboembolism [41]. It is estimated that 50–70% of patients with inadequate response to csDMARDs and bDMARDs achieve a good response to treatment with tsDMARDs [35]. Analyses of an RA cohort from the BIOBADASER III registry found that 24% of patients discontinued treatment with b/tsDMARDs due to adverse events [42]. This review aims to summarise current treatment practices of rheumatoid arthritis and highlight the pressing need for alternative approaches to new drug discovery by elucidating the relationships between drugs, genes and disease pathology and ultimately developing further effective and safe therapeutic options. This review discusses how computational approaches such as connectivity mapping can identify existing FDA-approved drugs that can be repurposed to be beneficial in alternate disease areas beyond their original treatment indication. This paper will overview the challenges and limitations of this approach as well as the opportunities and potential benefits.

## 2. Discussion

### 2.1. Limitations of Current Guidelines

There is a significant impact on quality of life and work capacity for individuals with RA. Studies evidence that the functional capacity of people living with RA deteriorates with time as disease activity increases [43]. There is also 15–46% increased mortality in people living with rheumatoid arthritis compared to the general population. Around one in six people have major depressive disorder, which is associated with increased levels of pain and function. Within the first year of diagnosis, 30–50% of people have to remove themselves from the workplace, which can become a permanent inability to work, leading to personal financial problems. Approximately 68% of RA patients in the UK are physically inactive, which becomes a vicious cycle of disease progression and increased pain, thus affecting both physical and mental health [44].

It is therefore clear that no drug is effective in every patient, and there is significant variability and overlap between treatments in terms of response and toxicity. Some patients are non-responsive to the treatment while experiencing side effects that can be severely detrimental to the patient’s health and quality of life. Another important factor contributing to treatment failure and is often overlooked is drug resistance caused by poorly understood underlying biological mechanisms.

Clinicians in the UK currently work on guidelines set by NICE, EULAR and the British Society of Rheumatology by cycling through the various drug combinations from the csDMARDs via biologic DMARDs to new targeted synthetic DMARDs. This approach is essentially a trial-and-error prescribing approach with minimal new guidance in instances of non-response other than that emerging from ongoing fundamental mechanistic research and clinical trials to resolve which combinations are effective and safe.

Studies report that although T2T is recommended as a standard practice of care and some aspects of the strategy are widely used, its full implementation remains uncommon, and adherence to the approach is low [45]. This is mainly due to a lack of knowledge and limited resources among healthcare providers on how to determine a suitable target and treatment for individual patients of such a complex disease that requires a multi-faceted treatment approach [46,47]. Despite the advancements and enhanced knowledge gained from innovative research, the translation of these into therapeutic benefits has not been fully realised. A study in Brazil found the average time lag from the clinical development to the application of biological drugs was 11.3 years [48]. Upadacitinib is the most recent treatment approved for RA with clinical trials to determine safety and efficacy in 2015 and approved for use in the UK in April 2021 [49].

Drug development is a time-consuming and expensive process with many pitfalls for pharmaceutical companies. A study assessing 640 phase 3 trials found a 54% failure rate due to reasons such as inadequate efficacy, concerns with safety and lack of funding [50]. The cost to successfully complete drug development to bring a drug to market is estimated at approximately $2.5 billion [51]. Therefore, failure at a later stage of the process results in substantial financial loss, which could have been spent pursuing another potential therapeutic candidate.

There is therefore a prescient need for alternative drug discovery approaches that exploit relationships between drugs, genes and disease pathways to identify alternate therapeutic candidates with efficacy and safety profiles suited to heterogeneous diseases such as RA. Clinicians require a reliable approach to efficiently identify and administer a highly effective therapy that can minimise disease activity, reduce disease burden and is also cost-effective. Pharmaceutical companies increasingly require discovery approaches with the potential to increase confidence in drug candidates ahead of clinical trials and to reduce the associated development timescales and costs by prioritising compounds or molecules with enhanced precision and efficacy.

### 2.2. Connectivity Mapping

Connectivity mapping (CMap) is a bioinformatic approach pioneered by Lamb et al. in 2006 with the basic concept of comparing a reference database of drug-related gene expression profiles with a query gene signature specific to a disease or a response to treatment in a disease [52]. This allows for the identification of associations between drugs and disease-related genes with the ultimate aim of predicting potential therapeutic options effective in that disease. Applications of CMap in pharmacogenomics include the discovery of novel phenotypic relations, elucidation of drug mechanism of action, drug repurposing and identification of drug combinations [52].

CMapBatch is a parallel approach to connectivity mapping adapted by Fortney et al. [53]. This approach is similar to meta-analysis as it applies CMAP to multiple gene signatures for the same disease and then combines the resulting outcomes [53]. Analysis of lung cancer data revealed that CMapBatch produces a more stable list of drugs when compared to individual gene signatures. Despite the fact that CMapBatch was only tested for lung cancer, the proposed meta-analysis can be used for any disease phenotype to prioritise therapeutics. For example, multiple colorectal cancer datasets were analysed to compile a gene signature consisting of 148 genes. CMap analysis with this signature identified 10 candidate compounds, including existing chemotherapies such as irinotecan and etoposide [54,55]. Other studies utilising CMap show promise by identifying candidate compounds and combination therapies for the treatment of breast cancer [56] and gastric cancer [57].

The CMap approach has been utilised for putative drug target investigations in autoimmune diseases. A study on Hashimoto’s thyroiditis performed CMap analysis on a human thyroid microarray dataset and found a causal link between viral infection and triggering or exacerbating the autoimmune response in the thyroid gland [58]. Comparisons of the disease gene signature against a perturbation gene expression database revealed potential markers and candidate drugs as promising therapeutics for the condition. Another study in multiple sclerosis, a complex inflammatory disease involving multiple disease pathways, used the CMap approach to analyse immune cell changes in transcriptomic datasets to identify potential target genes and candidate drugs from the CMAP database and DrugBank database that can be repositioned to engage multiple treatment pathways [59]. Cystic fibrosis and Huntington’s disease studies have validated the effectiveness of the CMap approach to identify small molecules with the potential to inhibit the disease state or regulate the expression of a small number of genes. For instance, A20 was identified as a key target to downregulate the pro-inflammatory NF-kB pathway, and the connectivity mapping approach predicted ikarugamycin and quercetin, FDA-approved drugs with anti-inflammatory effects, to induce A20 expression and therefore reduce the inflammatory response in cystic fibrosis [55]. Deferoxamine and chlorzoxazone, FDA-approved antioxidant and anti-inflammatory agents, were identified to reduce mutant HTT toxicity and HTT-induced caspase activation in PC12 cells, which can delay the onset or progression of Huntington’s disease [60].

### 2.3. Drug Repurposing and Sensitisation

Drug repurposing is a concept that has attracted considerable attention in recent years. The term drug repurposing is broadly defined as investigating drugs which are already approved for specific disease indications but may have utility in alternate diseases. The established safety profile of such drugs is a significant advantage, in addition to bypassing the time and cost involved with the *de novo* development of new compounds.

Monoclonal antibody treatments such as tocilizumab and mavrilimumab have been repurposed for use in COVID-19 and have been associated with reducing the incidence of severe infections and decreasing the duration of vasopressor support needed in severe patients. Studies on mavrilimumab concluded it was associated with improved clinical outcomes for severe COVID-19 patients with systemic hyperinflammation and pneumonia. JAK inhibitor, baricitinib, speeds up viral clearance and augments patients’ discharge rates compared to COVID-19 patients who have standard-of-care. A recent randomised clinical trial with 1033 patients showed a better therapeutic outcome of combined therapy of baricitinib with remdesivir for COVID-19 hospitalised patients compared to only remdesivir [61]. This highlights that the majority of recent research conducted in drug repurposing has focused on finding drugs that could combat the effects of the SARS-CoV-2 virus infection. Therefore, there is now a unique opportunity to apply similar principles in RA, a chronic and frequent treatment-refractory disease, to identify effective treatments which reduce disease activity and disease burden. On the other hand, the principle of drug sensitisation is when a drug exhibits synergistic effects with another drug to produce enhanced anti-disease efficacy that could not be achieved by using either drug in isolation [62]. The rationale behind this combined therapeutic approach is to target more than one disease-associated pathway during treatment. Such an approach is built on the premise that combination therapies simultaneously engage multiple pathways to evoke a higher response than those achieved with monotherapy. Another suggestion is that treatment with one drug can evoke a dynamic response, resulting in sensitivity to treatment with a second drug. It is believed that combinations of repurposed already approved drugs have good potential to achieve greater efficacy at lower dosages and may overcome drug resistance [63]. The implementation of synergistic combination therapy can raise concerns about synergistic toxicity as a result of targets and molecular mechanisms being shared between combined drugs. Rheumatology has widely adopted the concept of combination therapies, leading to improved outcomes in many cases [64]. However, there are multiple studies evidencing that drug combinations elevate the risk of adverse drug reactions. A study investigating adherence rates found that initiating and escalating combination treatment regimens resulted in increased adverse events, influencing drug discontinuation [65]. A study investigating the effects of hydroxychloroquine found that a combination with azithromycin significantly increased the risk of cardiovascular mortality [66]. These findings emphasise the need for careful consideration of drug–drug interactions and limitation of toxicity without compromising drug efficacy.

There are still many drugs routinely used for the treatment of RA which are yet to have their combinational effects fully explored.

### 2.4. Bioinformatics Pipelines to Identify Potential Therapeutics

Bioinformatic-led approaches are now more widely implemented within drug discovery pipelines for immune-mediated and inflammatory diseases. Recent studies illustrate the potential of bioinformatic approaches to exploit increasing volumes of data generated from clinical trials and studies carried out globally. Bioinformatic methods have been used to create data warehouses, algorithms, networks, and programs to analyse “big data” [67]. Drug development pipelines using bioinformatic resources and techniques have strong potential to accelerate candidate identification, avoid unwanted side effects and predict drug resistance [68].

For example, a drug discovery strategy was developed to identify potential therapeutic agents for inflammatory bowel disease. Data involving the NF-κB/RelA pathways were curated from multiple sources, including sequencing data, text-mining of relevant abstracts, genome-wide association studies and HumanPSD database [69]. Potential target genes within the pathways were classified as master regulators for pathway analysis. Prediction of activity spectra was used to assess the association between the chemical structure of compounds and their biological activities to identify potential novel drugs for inflammatory bowel disease treatment. Results of the study indicated that clarithromycin, a macrolide antibiotic, has the potential to act as an inhibitor of the NF-κB signalling pathway in the gastrointestinal tract. This finding complements existing clinical literature, as macrolides are already used to treat inflammatory conditions, such as panbronchiolitis [70] and atopic dermatitis [71]. The antibacterial and immunomodulatory properties of macrolides have shown promise in inhibiting the production and secretion of pro-inflammatory cytokines [71]. Additional studies investigating the effect of macrolide in combination with rifabutin for the treatment of Crohn’s disease indicate significant improvement in patient outcomes and disease activity [72]. This drug discovery approach incorporated an intentional bias towards target genes involved in the NF-κB signalling pathways, which resulted in corticosteroids and NSAIDs as the majority of predicted drugs.

An integrative computational modelling approach was developed to identify effective therapeutic agents for CD4+ T cell-mediated immune disorders. Multi-omic data was used to construct genome-scale metabolic models of CD4+ T cells to show perturbation in rheumatoid arthritis, multiple sclerosis, and primary biliary cholangitis. In silico simulations were performed on these models to predict drug targets from existing FDA-approved drugs and compounds with the potential to downregulate effector CD4+ T cells. Sixty-eight potential drug targets were identified and validated in vitro to propose several drugs that can be repurposed for RA, multiple sclerosis, and primary biliary cholangitis treatment [73].

### 2.5. Application of Artificial Intelligence in RA

Drug repurposing and use of artificial intelligence (AI) to accelerate discovery in legacy data is a concept that has garnered growing interest from pharmaceutical companies and research organisations in recent years, with several RA focussed studies proposing a computation-based drug discovery approach. One such study integrated drug-related and disease-related data to construct a genetic disease network to develop a drug-ranking algorithm. This algorithm discovered innovative drugs from diseases genetically related to RA that can be repositioned to treat RA [74]. A preclinical study demonstrated that repurposing of pirfenidone, a drug originally used to treat anti-pulmonary fibrosis, can inhibit inflammation and angiogenesis via multiple pathways in collagen-induced arthritic rats. This finding supports literature proposing the use of pirfenidone in RA; however, it requires further study in humans to reveal the potential of being used as a therapeutic in rheumatoid arthritis [75].

A further study used bioinformatic approaches to establish a transcriptional regulatory network to identify tissue-specific repurposing drug candidates for RA. The candidate drugs were reviewed and ranked based on supporting evidence obtained from extensive literature searches using text-mining analyses. Momelotinib, ibrutinib, and sodium butyrate were suggested as promising drug candidates, but further clinical studies are required to fully elucidate their therapeutic effects in patients with RA [76].

Medical image data also play a crucial role in understanding disease mechanism, progression and severity in RA. While image data is not commonly used as the primary input for AI-based drug discovery in RA, in recent years, data from various imaging techniques, such as MRI and ultrasound scans, have been used to develop algorithms and models to quantify synovitis and assess severity [75,77,78].

Numerous studies analysing treatment regimens in RA show that double and triple therapy leads to greater clinical outcomes than DMARD monotherapy. Combination therapy administered early in the course of the disease has been found to significantly decrease disease activity [79]. This finding aligns with the rationale behind the concept of drug sensitisation that administering multiple drugs in combination engages multiple pathways to evoke a higher treatment response. However, current evidence on combination therapy is limited, with knowledge gaps that can only be filled with further research and randomised controlled trials of adequate power.

The above studies demonstrate the power of implementing an in silico drug discovery model to identify repurposed candidate drugs and highlight the importance of incorporating steps to orthogonally validate results to determine which drugs to pursue for further experimental investigations.

**Figure 3:**
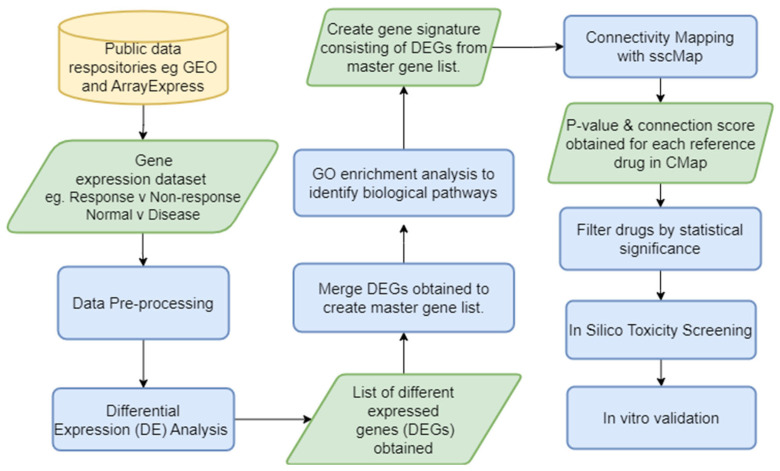
DrugExpress pipeline—In silico drug discovery and repurposing pipeline with an in vitro validation endpoint. GEO—Gene Expression Omnibus, DE—differential expression, DEG—differentially expressed genes, GO—gene ontology, sscMap—statistically significant connectivity map. Yellow cylinder represents the start point of the pipeline, green parallelogram represents input/output of a process and blue rectangle represents a process.

As an exemplar approach, we developed a novel bioinformatic pipeline (Figure 3), DrugExpress, which integrates the connectivity mapping platform sscMap (statistically significant connectivity map) [80] and ZhangScore [81,82]. DrugExpress will identify drug combinations, dosage regimens and already FDA-approved drugs that can be repurposed in rheumatoid arthritis. This pipeline also incorporates the novel concept of drug sensitisation by predicting drugs that will act as a sensitiser to another drug to produce synergistic effects which enhance therapeutic efficacy by targeting multiple disease pathways. Suitable candidate drugs will be identified and shortlisted based on their abilities to shift the transcriptomic (gene expression) profiles of treatment-naïve disease and to sensitise non-responding patient sub-groups towards more favourable “response-like” transcriptome profiles. Candidate drugs subsequently undergo toxicity screening and pathway analysis. The final list of candidate drugs then requires validation in vitro in RA model systems.

**Figure 4:**
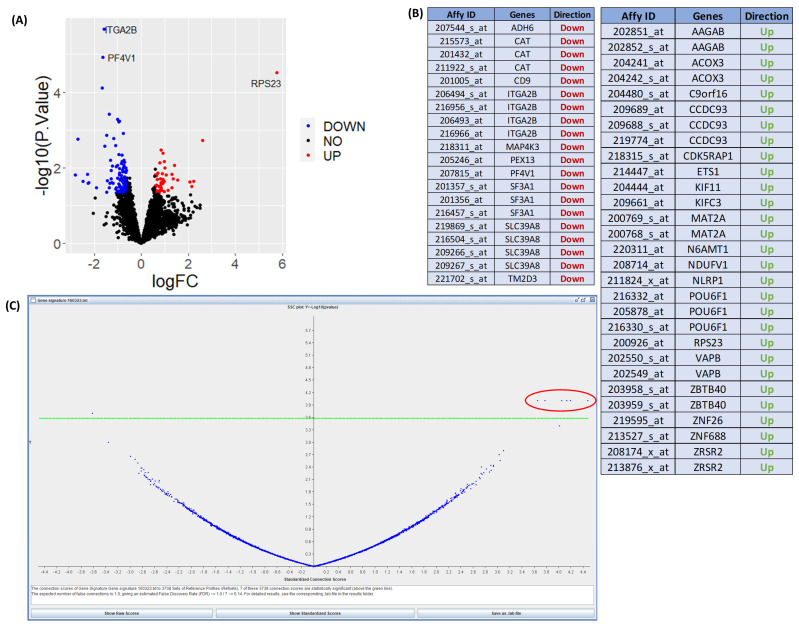
Results using the DrugExpress pipeline. (**A**) Volcano plot showing differentially expressed genes (labelled) between responders and non-responders to combined treatment of sulfasalazine and methotrexate from one suitable dataset. (**B**) Table of merged list of differentially expressed genes from multiple datasets. (**C**) Volcano plot of statistically significant candidate compounds located above the green threshold line with a perturbation stability score of 1. Statistically significant candidate drugs which would induce theoretical reduction in disease activity are located in the circled area of plot.

Figure 4 shows results from expression data after interrogation using the DrugExpress pipeline. Public datasets such as the Gene Expression Omnibus and ArrayExpress are mined to gather a collection of suitable datasets and pre-processed manually using Microsoft Excel and R programming. The datasets are filtered, sorted and selected based on the presence of disease activity scores, availability of clinical features, sample count and technological platform. Differential expression analysis was performed comparing response and non-response to treatment to obtain a list of differentially expressed genes (DEGs) characteristic of treatment response. DEGs from multiple datasets are merged to create a master gene list and subsequently mapped to Affymetrix probeset IDs to create a treatment response gene signature. Connectivity mapping (CMap) analysis is used to establish networks between DEGs in the response gene signature and FDA-approved drugs. *p*-value and connection scores of each reference drug in the CMap were obtained and used to determine statistical significance and perturbation stability. A total of six statistically significant candidate compounds were identified with a high probability of inducing therapeutic response. The next step is to perform in silico toxicity screening on the list of candidate compounds ahead of in vitro verification on optimal compounds to assess efficacy and ability to reduce gene expression in key pathways and cell proliferation associated with lower disease activity in MH7A human Ra synovial fibroblasts. This illustrates how publicly available expression datasets can be used to predict the theoretical effect of drug candidates and prioritise novel compounds with maximal potential to reduce disease activity.

### 2.6. Challenges and Benefits

Several challenges or limitations need to be acknowledged with a computational drug discovery and AI approach. While the approach can be widely generalised for many human diseases and performed using multiple omics data, the limited availability of large, reliable data warehouses and high-quality datasets required for comprehensive analysis contributes to some limitations [73]. AI-based approaches rely heavily on high-quality datasets for training and validation. Obtaining diverse and reliable datasets that truly represent a given population remains a challenge to ensure predictions are accurate and without bias. Potential data noise generated from incorporating multiple different datasets may influence the performance of the pipeline. Issues such as varying annotation standards, formatting, storage, and access may also affect the analytical performance and results obtained [83]. Integration of AI into drug discovery also raises ethical concerns, such as data privacy and security, algorithmic biases and responsible use of AI-driven insights to benefit people and society [84].

Overcoming these challenges and limitations associated with the integration of AI in drug discovery requires a multifaceted approach. One strategy to address data limitations is to encourage collaborative data-sharing initiatives among research groups and pharmaceutical companies to create larger, more diverse and representative datasets [85]. Initiatives to establish and implement data standardisation measures for data collection and annotation can ensure consistency and reliability when generating results [86]. Ethical considerations demand the establishment of ethics review boards and teams that conduct regular audits to minimise biases and implement strict data privacy and access protocols [84].

There are many limitations associated with implementing pharmacogenomic approaches in a clinical setting. The complexity of genetic variability poses challenges in interpreting the combined effects of multiple genes on drug response. Limited clinical evidence for the impact of specific genetic variations can hinder the broad applicability of pharmacogenomic findings [87]. Lifestyle and environmental factors interacting with genetic factors can also affect drug metabolism and response, particularly in the context of multiple combined medications, adding another layer of complexity [88]. Another challenge is the costs associated with the integration of pharmacogenomic testing into routine clinical practice, including the need for investment in training and support to bridge the knowledge gap [89]. Concerns relating to patient acceptance and compliance are another limitation of this approach.

On the other hand, potential benefits of AI-based methods include the identification of novel drug combinations, dosage regimens and application of already established treatments in a new index disease that can be used as priority candidates for further in vitro and in vivo biological validation [83].

## 3. Conclusions

RA is a disorder with considerable heterogeneity in disease severity and trajectory, which affects the accuracy of a patient’s prognosis and prescribing.

It is a chronic debilitating disorder that affects a significant proportion of the global population, which, when refractory to the current therapies, leads to a considerable societal, clinical and financial burden. It is therefore important that novel effective therapies are identified, trialled and approved more efficiently to help reduce the growing clinical and financial burden associated with poor treatment efficacy. This review overviews the substantial opportunity AI approaches present to incorporate disease-related and drug-related data to streamline the development of new treatments with favourable outcomes.

## Data Availability

Publicly available datasets were analysed in this study. This data can be found here: https://www.ncbi.nlm.nih.gov/geo/. Accession number: GSE93777, GSE24742, GSE97948.
